# Tailoring study design to each stage of surgical innovation: the ideal recommendations

**DOI:** 10.1186/1745-6215-14-S1-O85

**Published:** 2013-11-29

**Authors:** Allison Hirst, Jonathan A Cook, Peter McCulloch, Douglas G Altman, Carl Heneghan, Markus K Diener, Patrick L Ergina, Jeffrey S Barkun, Jane M Blazeby, David J Beard, Danica Marinac-Dabic, Art Sedrakyan

**Affiliations:** 1 Nuffield Department of Surgical Science, University of Oxford, Oxford, UK; 2 Health Services Research Unit, University of Aberdeen, Aberdeen, UK; 3 Nuffield Department of Surgical Science, University of Oxford, John Radcliffe Hospital, Oxford, UK; 4 Centre for Statistics in Medicine, University of Oxford, Botnar Research Centre, Oxford, UK; 5 Department of Public Health and Primary Care, Oxford University, Oxford, UK; 6 Director of the Study Centre of the German Surgical Society, Department of General-, Visceral-, and Transplantation Surgery, Heidelberg University, Heidelberg, Germany; 7 Department of Surgery, McGill University, and Oxford International Programme in Evidence-Based Health Care, University of Oxford, Montreal, Canada; 8 Department of Surgery, McGill University, Montreal, Canada; 9 Professor of Surgery, Centre for Surgical Research, School of Social & Community Medicine, University of Bristol, Bristol, UK; 10 Nuffield Department of Orthopaedics, Rheumatology and Musculoskeletal Sciences, University of Oxford, and NIHR Oxford Musculoskeletal Biomedical Research Unit, Oxford, UK; 11 Division of Epidemiology, Office of Surveillance and Biometrics, Center for Devices and Radiological Health, Food and Drug Administration, Maryland, USA; 12 Weill Cornell Medical College of Cornell University and New York Presbyterian Hospital, New York City, NY, USA

## 

The pathway of surgical innovation is complex. Inherent ethical and practical characteristics make scientific evaluation of new techniques or devices by a definitive randomized controlled trial (RCT) challenging.

The IDEAL Collaboration (http://www.ideal-collaboration.net) Framework for evaluating surgical innovation describes a five stage process - **I**dea, **D**evelopment, **E**xploration, **A**ssessment and **L**ong-term study.^(1)^ Early stage studies should be designed to facilitate and prepare the way for a rigorous evaluation by RCT.

IDEAL Recommendations in the early stages (**I**dea/**D**evelopment) emphasise prospective designs, transparency and full reporting in open registries, to provide reliable data early in the innovation development process. At the **E**xploration stage, prospective observational studies need to address factors such as case-mix, learning and outcomes, building co-operatively and explicitly towards a definitive evaluation study, preferably an RCT, optimising the contribution of data from non-randomised prospective evaluations (**A**ssessment stage). The **L**ong-term stages should be characterised by registry-based surveillance for both new procedures and devices.

IDEAL proposals for high quality RCTs of surgical procedures focus on three key areas: definition of the intervention; who delivers the intervention and preferences of surgeons and patients. IDEAL Recommendations identify modifications to study design which may help address these difficult areas. We will describe examples of good practice using these suggested methods.

Everyone involved in evaluating surgical innovations is invited to join the IDEAL Collaboration community and help further evolve methodology and reporting standards for robust trials in surgery.
